# Organic synthesis using (diacetoxyiodo)benzene (DIB): Unexpected and novel oxidation of 3-oxo-butanamides to 2,2-dihalo-*N*-phenylacetamides

**DOI:** 10.3762/bjoc.8.38

**Published:** 2012-03-07

**Authors:** Wei-Bing Liu, Cui Chen, Qing Zhang, Zhi-Bo Zhu

**Affiliations:** 1 School of Chemistry and Life Science, Guangdong University of Petrochemical Technology, Maoming 525000, China; 2 College of Pharmaceutical Sciences, Southern Medical University, Guangzhou 510515, China

**Keywords:** cleavage of carbon–carbon bond, (diacetoxyiodo)benzene, difunctionalized acetamides, novel oxidation, 3-oxo-*N*-phenylbutanamides

## Abstract

A novel and reliable method for the direct preparation of 2,2-dihalo-*N*-phenylacetamides is reported. The key transformation involves the cleavage of a carbon–carbon bond in the presence of DIB and a Lewis acid as the halogen source, and thus this method significantly expands the value of DIB as a unique and powerful tool in chemical synthesis. This protocol not only adds a new aspect to reactions that use other hypervalent iodine reagents but also provides a wide space for the synthesis of disubstituted acetamides.

## Introduction

**Scheme 1:**
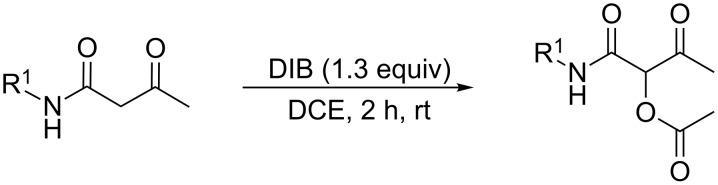
Synthesis of 1-carbamoyl-2-oxopropyl acetates.

Hypervalent iodine(III) reagents [1–18] have received much attention, as reflected by the plethora of publications and reviews [19–23]. This is due to their low toxicity, ready availability, easy handling, clean transformation, and reactivity, which is similar to heavy-metal-based oxidants, including harmful elements, such as Pb(IV), Hg(II), and Tl(III), as well as transition metal-catalyzed processes [24–30]. Recently, we reported an efficient acetoxylation approach to synthesize 1-carbamoyl-2-oxopropyl acetate derivatives by using (diacetoxyiodo)benzene (DIB) (Scheme 1) [31].

**Scheme 2:**
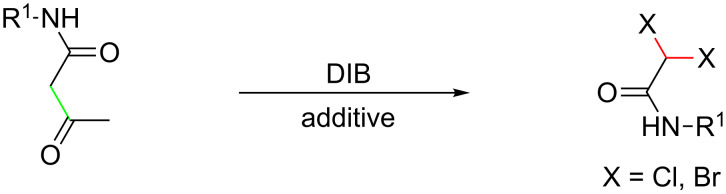
Synthesis of 2,2-dihalo-*N*-phenylacetamides.

During the course of conditional optimization to synthesize 1-carbamoyl-2-oxopropyl acetate derivatives, we surprisingly found that almost none of the desired acetoxylation product was obtained, but 2,2-dichloro-*N*-phenylacetamide was provided as the major product, upon addition of Lewis acids such as FeCl_3_, ZnCl_2_ and CuCl_2_ in the reaction system. Based on this result, we developed a simple and efficient approach to the synthesis of 2,2-dihalo-*N*-phenylacetamides, on which we report herein (Scheme 2). To the best of our knowledge, there are several reports on chlorination and bromination reactions with PhI(OAc)_2_ and a halogen source such as TMSBr, lithium halide or pyridinium halide [32–34]. Also, there are several reports on the synthesis of difunctionalized acetamide derivatives [35–38], but this report is the first to describe the synthesis of 2,2-dihalo-*N*-phenylacetamides through an oxidative process with PhI(OAc)_2_ and Lewis acids as the halogen source.

## Results and Discussion

Initially, we used 3-oxo-*N*-phenylbutanamide (**1a**) as the model substrate to optimize the reaction conditions in different solvents, temperatures and amounts of DIB (Table 1). The best result was obtained in dioxane in the presence of 1.3 equiv of DIB and 1.5 equiv of zinc(II) chloride at room temperature for one hour (Table 1, entry 11). For this transformation, FeCl_3_ and ZnCl_2_ were suitable Lewis acids (Table 1, entry 2 and entry 3), and dioxane and DMF were practical solvents among the various solvents examined (Table 1, entry 3 and entry 8). It is noteworthy that no product **2a** was obtained when the reaction was carried out without the addition of Lewis acids (Table 1, entry 1) or without DIB (Table 1, entry 5).

**Table 1:** Optimization of reaction conditions.^a^

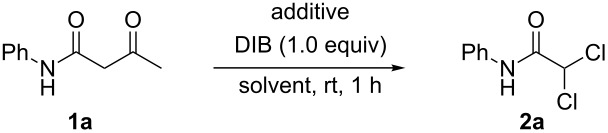				

entry	solvent	additive (1.5 equiv)	time (h)	yield (%)^b^

1	dioxane	none	1	–
2	dioxane	FeCl_3_	1	78
3	dioxane	ZnCl_2_	1	81
4^c^	dioxane	ZnCl_2_	1	75
5^d^	dioxane	ZnCl_2_	1	–
6	cyclohexane	ZnCl_2_	1	26
7	DCE	ZnCl_2_	1	42
8	DMF	ZnCl_2_	1	80
9	DMSO	ZnCl_2_	1	46
10^e^	dioxane	ZnCl_2_	1	31
11^f^	dioxane	ZnCl_2_	1	89
12^g^	dioxane	ZnCl_2_	1	84
13^f^	dioxane	ZnCl_2_	0.5	53
14^f^	dioxane	ZnCl_2_	1.5	89
15^f^	dioxane	ZnCl_2_	2	89

^a^**1a** (0.25 mmol), solvent (2 mL), DIB (1.0 equiv); ^b^GC yield; ^c^ZnCl_2_ (1.0 equiv); ^d^without DIB; ^e^DIB (0.5 equiv); ^f^DIB (1.3 equiv); ^g^DIB (2.0 equiv).

**Scheme 3:**
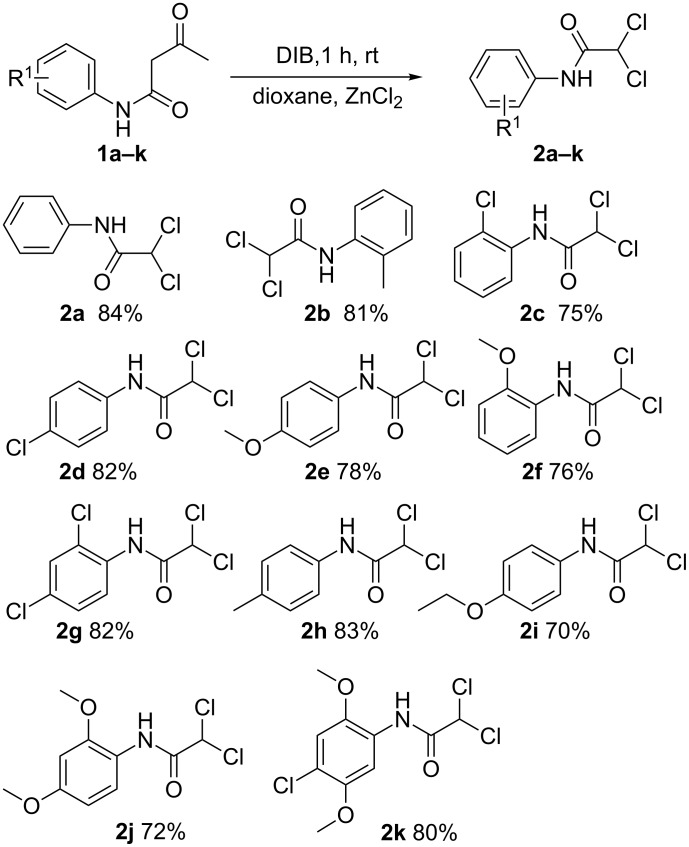
Synthesis of dichloroacetamides. Reagents and conditions: **1** (1.0 mmol), dioxane (2 mL), DIB (1.3 equiv), ZnCl_2_(1.5 equiv); yields % are isolated yields.

After optimizing the reaction conditions, we used a range of 3-oxo-*N*-phenylbutanamides to explore the substrate scope and limitations of this reaction. As shown in Scheme 3, all the reactions proceeded smoothly and gave the corresponding *N*-phenyl dichloroacetamides **2a**–**2k** exclusively and in good to excellent isolated yields. It was also found that the number and the electronic properties of the substituents on the benzene ring had little effect on the reaction. For example, the reactions of 3-oxo-*N*-phenylbutanamide (**1a**), 3-oxo-*N*-*o*-tolylbutanamide (**1b**), *N*-(2,4-dimethoxyphenyl)-3-oxobutanamide (**1j**) and *N*-(4-chloro-2,5-dimethoxyphenyl)-3-oxobutanamide (**1k**) all led to their corresponding *N*-phenyl dichloroacetamides (Scheme 3, **2a**, **2b**, **2j**, and **2k**) in good isolated yields. In addition, the position of the substituents on the benzene ring also has little effect on this transformation, such that *N*-(2-chlorophenyl)-3-oxobutanamide (**1c**), *N*-(4-chlorophenyl)-3-oxobutanamide (**1d**), *N*-(4-methoxyphenyl)-3-oxobutanamide (**1e**) and *N*-(2-methoxyphenyl)-3-oxobutanamide (**1f**) could also serve as good substrates in this protocol (Scheme 3, **2c**, **2d**, **2e**, and **2f**).

**Scheme 4:**
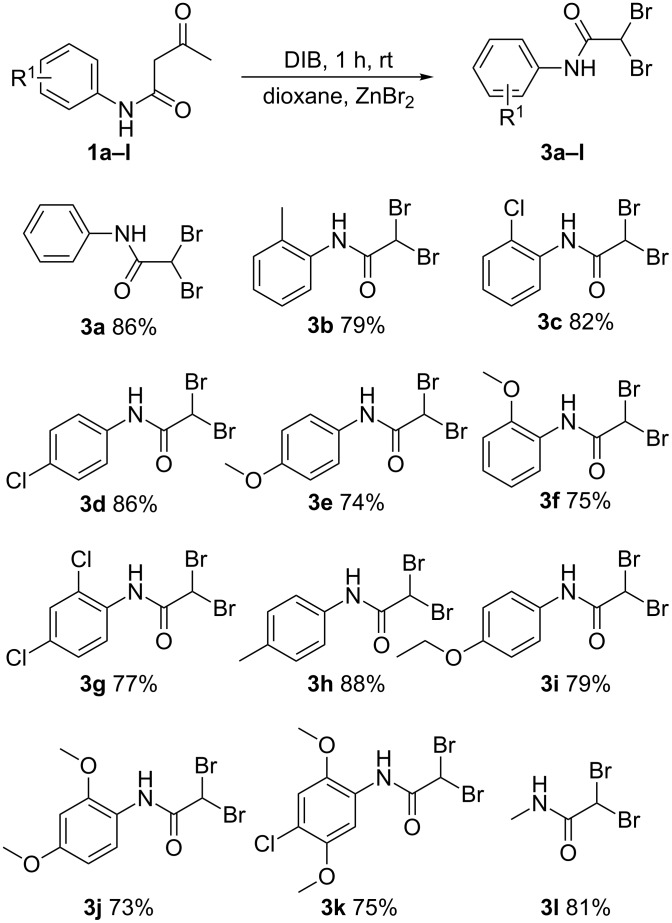
Synthesis of dibromoacetamides. Reagents and conditions: **1** (1.0 mmol), dioxane (2 mL), DIB (1.3 equiv), ZnBr_2_ (1.5 equiv); yields % are isolated yields.

Next, in order to expand the scope of this protocol, we employed ZnBr_2_ as a reagent under the same reaction conditions, and we were pleased to find that the corresponding dibromo derivatives were obtained as the products. As shown in Scheme 4, all tested substrates provided the corresponding dibromoacetamides **3a**–**3l** in good to excellent isolated yields, which not only greatly expanded the application scope of this protocol but also provided a wide space for the synthesis of 2,2-dihalo-*N*-phenylacetamides.

**Scheme 5:**
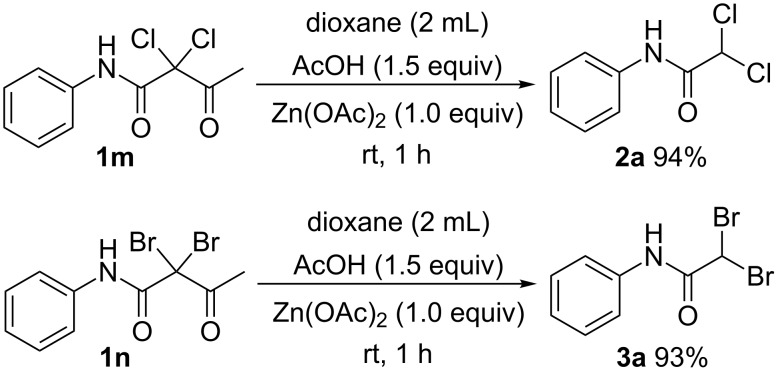
Probe the mechanism.

In spite of the widespread use of DIB, there is no direct precedent for DIB-mediated cleavage of C–C bonds. In particular, the application of this protocol to synthesize difunctionalized acetamides from 3-oxo-butanamides is reported here for the first time. In order to probe the mechanism of this transformation, we employed 2,2-dichloro-3-oxo-*N*-phenylbutanamide (**1m**) and 2,2-dibromo-3-oxo-*N*-phenylbutanamide (**1n**) as reactants under acidic conditions in the presence of Zn(OAc)_2_ (Scheme 5), and we found that the reaction can also give the corresponding product 2,2-dichloro-*N*-phenylacetamide (**2a**) and 2,2-dibromo-*N*-phenylacetamide (**3a**).

**Scheme 6:**
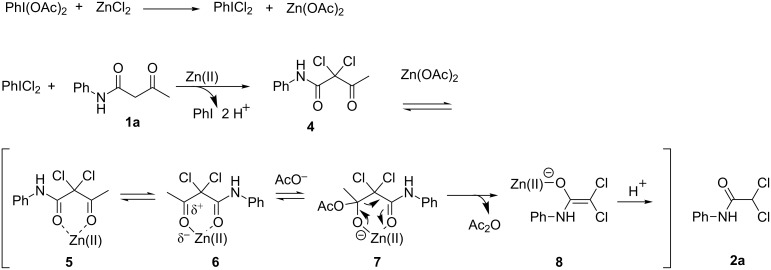
Tentative mechanism for the synthesis of 2,2-dihalo-*N*-phenylacetamides.

On the basis of these preliminary results, a mechanistic proposal for this transformation, exemplified by the formation of **2a**, is depicted in Scheme 6. Initially, the reaction involved generation of the known chlorinating agent (dichloroiodo)benzene (PhICl_2_) [39], followed by dichlorination of the β-keto amide of 3-oxo-*N*-phenylbutanamide (**1a**) to give intermediate **4**. It is well known that Lewis acids can activate 1,3-diketones [40] to produce intermediate **5** and **6**. This complexation not only increases the nucleophilicity of the methylene carbon atom, but also simultaneously increases the electrophilicity of the carbonyl carbon atom. Consequently, nucleophilic attack of the acetate ion on the carbonyl carbon atom affords intermediate **7**. A subsequent carbon–carbon bond cleavage of the labile α,α-dichloro β-keto amide through a retro-Claisen condensation reaction [41] generates intermediate **8**. Finally, the electrophilic attack of a proton on the carbon–carbon double bond resulted in the final product 2,2-dichloro-*N*-phenylacetamide (**2a**).

## Conclusion

In summary, we have shown an efficient and operationally simple method to synthesize 2,2-dihalo-*N*-phenylacetamides. The mild reaction conditions, good substrate scope and good to excellent yields make the present protocol potentially useful in organic synthesis. Moreover, it should be pointed out that this transformation includes an oxidative process involving the cleavage of a carbon–carbon bond, which significantly expands the value of DIB as a unique and powerful tool in chemical synthesis. Future studies on the application of this protocol to the synthesis of other difunctionalized acetamides and detailed investigations of the reaction mechanism are in progress.

## Supporting Information

File 1 Experimental details and characterization of compounds.
