# Supplementary material for: Organic synthesis using (diacetoxyiodo)benzene (DIB): Unexpected and novel oxidation of 3-oxo-butanamides to 2,2-dihalo-N-phenylacetamides
Source: Beilstein J Org Chem. 2012 Mar 7;8:344–8. doi: 10.3762/bjoc.8.38 (PMC3326611; doi:10.3762/bjoc.8.38)
Supplement: File 1 — Experimental details and characterization of compounds. [file Beilstein_J_Org_Chem-08-344-s001.pdf]

Supporting Information

for

Organic synthesis using (diacetoxyiodo)benzene (DIB): Unexpected and novel oxidation of 3-oxo-butanamides to 2,2-dihalo-*N*-phenylacetamides

Wei-Bing Liu*[1], Cui Chen[1], Qing Zhang[1] and Zhi-Bo Zhu*[2]

Address: [1]School of Chemistry and Life Science, Guangdong University of Petrochemical Technology, 2 Guangdu Road, Maoming 525000, China and [2]College of Pharmaceutical Sciences, Southern Medical University, Guangzhou 510515, China

E-mail: Wei-Bing Liu - lwb409@yahoo.com.cn; Zhi-Bo Zhu - zzb24@yahoo.com.cn

*Corresponding author

Experimental details and characterization of compounds.

General method: All reactions were carried out at room temperature in a Schlenk tube equipped with a magnetic stir bar. Solvents and all reagents were used as received. ^{1}H NMR spectra were recorded in $CDCl_3$ at 400 MHz and ^{13}C NMR spectra were recorded in $CDCl_3$ at 100 MHz. GC–MS was obtained by using electron ionization (EI). TLC was performed on commercially prepared 100–400 mesh silica gel plates (GF_{254}), and visualization was effected at 254 nm. All other chemicals were purchased from Aldrich Chemicals.

Typical procedure for the synthesis of 2,2-dichloro-*N*-phenylacetamide (2a): To a 10 mL Schlenk tube was added DIB (419 mg, 1.3 mmol), dioxane (2 mL), 3-oxo-*N*-phenylbutanamide (**1a**) (177 mg, 1.0 mmol) and $ZnCl_2$ (204 mg, 1.5 mmol). The mixture was stirred at rt for 1 h. The solution was directly subjected to isolation by PTLC (GF254), eluted with cyclohexane, which furnished **2a** (171.4 mg, 84%) as a white solid.

Typical procedure for the synthesis of 2,2-dibromo-*N*-phenylacetamide (3a): To a 10 mL Schlenk tube was added DIB (419 mg, 1.3 mmol), dioxane (2 mL), 3-oxo-*N*-phenylbutanamide (**1a**) (177 mg, 1.0 mmol) and $ZnBr_2$ (334 mg, 1.5 mmol). The mixture was stirred at rt for 1 h. The solution was directly subjected to isolation by PTLC (GF254), eluted with cyclohexane, which furnished **3a** (251.2 mg, 86%) as a colorless solid.

Typical procedure for the synthesis of 2,2-dichloro-*N*-phenylacetamide (Scheme 5, 2a): To a 10 mL Schlenk tube was added dioxane (2 mL), 2,2-dichloro-3-oxo-*N*-phenylbutanamide (**4**) (61.5 mg, 0.25 mmol), $Zn(OAc)_2$ (46 mg, 0.25 mmol) and AcOH (23 mg, 0.375 mmol). The mixture was stirred at rt for 1 h. The solution was directly subjected to isolation by PTLC (GF254), eluted with cyclohexane, which furnished **2a** (48 mg, 94%) as a white solid.

2,2-Dichloro-*N*-phenylacetamide (2a) [1]

Colorless solid; mp 121–123 °C; IR (KBr) ν_{max}: 3273, 1691, 1600, 1544, 1442, 1298, 1174, 968, 860, 810, 756 cm^{-1}; ^{1}H NMR ($CDCl_3$, 400 MHz) δ 8.22 (s, 1H), 7.55–7.53 (d, *J* = 8.0 Hz, 2H), 7.37–7.33 (t, 2H), 7.20–7.16 (t, 1H), 6.04 (s, 1H); ^{13}C NMR ($CDCl_3$, 100 MHz) δ 162.9, 136.5, 129.4, 125.9, 120.5, 67.1; EIMS *m*/*z* (%): 120.05 (100.00), 202.95 (26.81).

2,2-Dichloro-*N*-*o*-tolylacetamide (2b) [1]

White solid; mp 140–142 °C; IR (KBr) ν_{max}: 3253, 1672, 1546, 1450, 1246, 1114, 974, 871, 806, 756 cm^{-1}; ^{1}H NMR ($CDCl_3$, 400 MHz) δ 8.09 (s, 1H), 7.74–7.72 (d, *J* = 8.0 Hz, 1H), 7.24–7.20 (m, 2H), 7.16–7.12 (m, 1H), 6.05 (s, 1H), 2.29 (s, 3H); ^{13}C NMR ($CDCl_3$, 100 MHz) δ 162.2, 134.2, 130.9, 130.2, 127.2, 126.6, 123.2, 67.2, 17.6; EIMS *m*/*z* (%): 134.05 (100.00), 216.90 (28.02).

2,2-Dichloro-*N*-(2-chlorophenyl)acetamide (2c) [1]

White solid; mp 115–117 °C; IR (KBr) ν_{max}: 3250, 1678, 1595, 1541, 1435, 1174, 1041, 804, 752 cm^{-1}; ^{1}H NMR ($CDCl_3$, 400 MHz) δ 8.79 (s, 1H), 8.30–8.28 (d, *J* = 8.0 Hz, 1H), 7.41–7.39 (d, *J* = 8.0 Hz, 1H), 7.32–7.28 (m, 1H), 7.14–7.09 (m, 1H), 6.06 (s, 1H); ^{13}C NMR ($CDCl_3$, 100 MHz) δ 161.9, 133.4, 129.5, 128.1, 126.2, 124.0, 121.6, 67.1; EIMS *m*/*z* (%): 154.00 (100.00), 236.85 (20.86).

2,2-Dichloro-*N*-(4-chlorophenyl)acetamide (2d) [1]

Colorless solid; mp 140–142 °C; IR (KBr) ν_{max}: 3280, 1680, 1605, 1546, 1487, 1396, 1232, 1093, 813, 746, 684 cm^{-1}; ^{1}H NMR ($CDCl_3$, 400 MHz) δ 8.18 (s, 1H), 7.50–7.48 (d, *J* = 8.0 Hz, 2H), 7.33–7.31 (d, *J* = 8.0 Hz, 2H), 6.03 (s, 1H); ^{13}C NMR ($CDCl_3$, 100 MHz) δ 162.1, 135.1, 129.6, 121.8, 67.0; EIMS *m*/*z* (%): 153.95 (100.00), 236.80 (25.80).

2,2-Dichloro-*N*-(4-methoxyphenyl)acetamide (2e) [1]

Colorless solid; mp 135–137 °C; IR (KBr) ν_{max}: 3285, 1685, 1602, 1516, 1455, 1247, 1174, 1029, 831, 798 cm^{-1}; ^{1}H NMR ($CDCl_3$, 400 MHz) δ 8.07 (s, 1H), 7.42–7.44 (d, *J* = 8.0 Hz, 2H), 6.89–6.84 (q, 2H), 6.01 (s, 1H), 3.78 (s, 1H); ^{13}C NMR ($CDCl_3$, 100 MHz) δ 160.7, 129.1, 128.9, 122.1, 114.3, 66.8, 55.4; EIMS *m*/*z* (%): 150.00 (100.00), 232.85 (36.70).

2,2-Dichloro-*N*-(2-methoxyphenyl)acetamide (2f)

Orange solid; mp 85–87 °C; IR (KBr) ν_{max}: 3280, 1680, 1615, 1541, 1450, 1382, 1114, 1022, 866, 198, 650 cm^{-1}; ^{1}H NMR ($CDCl_3$, 400 MHz) δ 8.86 (s, 1H), 8.29–8.24 (m, 1H), 7.13–7.11 (m, 1H), 6.97–6.91 (m, 1H), 6.91–6.89 (m, 1H), 6.02 (s, 1H), 3.91 (s, 1H); ^{13}C NMR ($CDCl_3$, 100 MHz) δ 161.3, 126.1, 125.5, 125.1, 121.1, 119.6, 110.1, 67.0, 55.9; EIMS *m*/*z* (%): 150.00 (100.00), 232.85 (36.70); Anal. calcd for $C_9H_9C_{12}NO_2$; C, 46.18; H, 3.88; N, 5.98; found: C, 46.25; H, 3.71; N, 6.17.

2,2-Dichloro-*N*-(2,4-dichlorophenyl)acetamide (2g)

Brown solid; mp 140–142 °C; IR (KBr) ν_{max}: 3275, 1685, 1585, 1541, 1450, 1259, 1093, 869, 810, 698 cm^{-1}; ^{1}H NMR ($CDCl_3$, 400 MHz) δ 8.77 (s, 1H), 8.38 (d, *J* = 8.0 Hz, 1H), 7.32–7.32 (d, *J* = 8.0 Hz, 1H), 7.11–7.08 (m, 1H), 6.05 (s, 1H); ^{13}C NMR ($CDCl_3$, 100 MHz) δ 161.6, 133.9, 133.7, 129.9, 125.9, 121.7, 121.2, 66.6; EIMS *m*/*z* (%): 235.85 (100.00), 272.80 (27.27); Anal. calcd for $C_8H_5Cl_4NO$; C, 35.20; H, 1.85; N, 5.13; found: C, 35.11; H, 1.99; N, 5.02.

2,2-Dichloro-*N*-*p*-tolylacetamide (2h) [1]

Colorless solid; mp 159–161 °C; IR (KBr) ν_{max}: 3242, 1704, 1672, 1517, 1406, 1242, 1176, 867, 810, 744 cm^{-1}; ^{1}H NMR ($CDCl_3$, 400 MHz) δ 8.15 (s, 1H), 7.43–7.41 (d, *J* = 8.0 Hz, 2H), 7.17–7.15 (d, *J* = 8.0 Hz, 2H), 6.03 (s, 1H), 2.33 (s, 1H); ^{13}C NMR ($CDCl_3$, 100 MHz) δ 161.6, 135.4, 133.6, 129.6, 120.2, 66.8, 20.8; EIMS *m*/*z* (%): 134.05 (100.00), 216.90 (30.31).

2,2-Dichloro-*N*-(4-ethoxyphenyl)acetamide (2i)

Pale yellow solid; mp 144–145; IR (KBr) ν_{max}: 3292, 2929, 1715, 1685, 1602, 1516, 1460, 1242, 1114, 1045, 921, 833, 802 cm^{-1}; ^{1}H NMR ($CDCl_3$, 400 MHz) δ 8.35 (s, 1H), 7.42–7.39 (q, 2H), 6.86–6.82 (m, 2H), 6.06 (s, 1H), 4.00–3.96 (q, *J* = 8.0 Hz, 2H), 1.39–1.35 (t, *J* = 8.0 Hz, 2H); ^{13}C NMR ($CDCl_3$, 100 MHz) δ 161.7, 156.5, 128.7, 122.0, 114.7, 66.8, 63.6, 14.6; EIMS *m*/*z* (%): 108.05 (100.00), 246.90 (47.09); Anal. calcd for $C_{10}H_{11}Cl_2NO_2$; C, 48.41; H, 4.47; N, 5.65; found: C, 48.56; H, 4.41; N, 5.59.

2,2-Dichloro-*N*-(2,4-dimethoxyphenyl)acetamide (2j)

Pale yellow solid; mp 107.0–108.0 °C; IR (KBr) ν_{max}:3295, 1683, 1610, 1535, 1462, 1282, 1122, 1033, 921, 805, 705 cm^{-1}; ^{1}H NMR ($CDCl_3$, 400 MHz) δ 8.66 (s, 1H), 8.17–8.15 (d, *J* = 8.0 Hz, 1H), 8.15–8.13 (d, *J* = 8.0 Hz, 1H), 6.46 (s, 1H), 6.01 (s, 1H), 3.87 (s, 3H), 3.78 (s, 3H); ^{13}C NMR ($CDCl_3$, 100 MHz) δ 160.9, 157.4, 149.7, 120.5, 119.6, 103.8, 98.7, 67.0, 55.9, 55.5; EIMS *m*/*z* (%): 152.05 (100.00), 262.90 (57.45); Anal. calcd for $C_{10}H_{11}Cl_2NO_3$; C, 45.48; H, 4.20; N, 5.30; found: C, 45.35; H, 4.16; N, 5.49.

2,2-Dichloro-*N*-(4-chloro-2,5-dimethoxyphenyl)acetamide (2k)

White solid; mp 111–113 °C; IR (KBr) ν_{max}: 3284, 2945, 1689, 1595, 1500, 1215, 1031, 970, 864, 804, 719 cm^{-1}; ^{1}H NMR ($CDCl_3$, 400 MHz) δ 8.82 (s, 1H), 8.09 (s, 1H), 6.91 (s, 1H), 6.03 (s, 1H), 6.01 (s, 1H), 3.85 (s, 6H), 3.78 (s, 3H); ^{13}C NMR ($CDCl_3$, 100 MHz) δ 161.4, 149.1, 142.4, 125.3, 117.4, 112.5, 104.7, 66.9, 56.7, 56.6; EIMS *m*/*z* (%): 198.90 (100.00), 296.85 (95.55); Anal. calcd for $C_{10}H_{10}Cl_3NO_3$; C, 40.23; H, 3.38; N, 4.69; found: C, 40.36; H, 3.29; N, 4.81

2,2-Dibromo-*N*-phenylacetamide (3a)

Colorless solid; mp 135–137 °C; IR (KBr) ν_{max}: 3273, 1691, 1600, 1544, 1442, 1298, 1174, 968, 860, 810, 756 cm^{-1}; ^{1}H NMR ($CDCl_3$, 400 MHz) δ 8.06 (s, 1H), 7.54–7.52 (d, *J* = 8.0 Hz, 2H), 7.39–7.35 (t, *J* = 8.0 Hz, 2H), 7.20–7.16 (t, *J* = 8.0 Hz, 1H), 5.90

(s, 1H); ^{13}C NMR ($CDCl_3$, 100 MHz) δ 168.2, 137.1, 129.2, 125.6, 120.0, 37.1; EIMS *m*/*z* (%): 120.05 (100.00), 292.70 (19.11); Anal. calcd for $C_8H_7Br_2NO$: C, 32.80; H, 2.41; N, 4.78; found: C, 31.91; H, 2.45; N, 4.90.

2,2-Dibromo-*N*-*o*-tolylacetamide (3b)

Colorless solid; mp 146–148 °C; IR (KBr) ν_{max}: 3273, 1691, 1600, 1544, 1442, 1298, 1174, 968, 860, 810, 756 cm^{-1}; ^{1}H NMR ($CDCl_3$, 400 MHz) δ 8.04 (s, 1H), 7.78–7.74 (d, *J* = 8.0 Hz, 2H), 7.26–7.20 (q, *J* = 8.0 Hz, 2H), 7.16–7.12 (t, *J* = 8.0 Hz, 1H), 5.93 (s, 1H); ^{13}C NMR ($CDCl_3$, 100 MHz) δ 170.9, 132.1, 130.2, 128.8, 128.6 124.9, 119.8, 36.9, 17.8; EIMS *m*/*z* (%): 134.00 (100.00), 306.70 (28.69); Anal. calcd for $C_9H_9Br_2NO$: C, 35.21; H, 2.96; N, 4.56; found: C, 35.02; H, 2.81; N, 4.66.

2,2-Dibromo-*N*-(2-chlorophenyl)acetamide (3c)

Colorless solid; mp 135.5–137 °C; IR (KBr) ν_{max}: 3273, 1691, 1600, 1544, 1442, 1298, 1174, 968, 860, 810, 756 cm^{-1}; ^{1}H NMR ($CDCl_3$, 400 MHz) δ 8.78 (s, 1H), 8.26–8.24 (d, *J* = 8.0 Hz, 1H), 7.42–7.40 (q, *J* = 8.0 Hz, 1H), 7.32–7.28 (t, *J* = 8.0 Hz, 1H), 7.11–7.09 (d, *J* = 8.0 Hz, 1H), 5.93 (s, 1H); ^{13}C NMR ($CDCl_3$, 100 MHz) δ 161.9, 133.4, 129.2, 127.8, 125.8, 123.8, 121.3, 36.4; EIMS *m*/*z* (%): 153.90 (100.00), 326.65 (24.49); Anal. calcd for $C_8H_6Br_2ClNO$: C, 29.35; H, 1.85; N, 4.28; found: C, 29.44; H, 1.99; N, 4.41.

2,2-Dibromo-*N*-(4-chlorophenyl)acetamide (3d)

Colorless solid; mp 140–141.5 °C; IR (KBr) ν_{max}: 3273, 1691, 1600, 1544, 1442, 1298, 1174, 968, 860, 810, 756 cm^{-1}; ^{1}H NMR ($CDCl_3$, 400 MHz) δ 8.07 (s, 1H), 7.49–7.47 (d, *J* = 8.0 Hz, 2H), 7.32–7.30 (t, *J* = 8.0 Hz, 1H), 5.90 (s, 1H); ^{13}C NMR ($CDCl_3$, 100 MHz) δ 162.3, 133.7, 129.2, 121.4, 121.3, 36.7; EIMS *m*/*z* (%): 291.70 (100.00), 326.65 (24.98); Anal. calcd for $C_8H_6Br_2ClNO$: C, 29.35; H, 1.85; N, 4.28; found: C, 29.24; H, 2.02; N, 4.22.

2,2-Dibromo-*N*-(4-methoxyphenyl)acetamide (3e)

Tan oil; IR (KBr) ν_{max}: 3273, 1691, 1600, 1544, 1442, 1298, 1174, 968, 860, 810, 756 cm^{-1}; ^{1}H NMR ($CDCl_3$, 400 MHz) δ 8.06 (s, 1H), 7.44–7.42 (d, *J* = 8.0 Hz, 2H), 6.90–6.88 (d, *J* = 8.0 Hz, 2H), 5.91 (s, 1H), 3.79 (s, 3H); ^{13}C NMR ($CDCl_3$, 100 MHz) δ 162.0, 156.3, 129.4, 122.0, 114.3, 55.5, 36.8; EIMS *m*/*z* (%): 149.11 (100.00), 323.04 (9.88); Anal. calcd for $C_9H_9Br_2NO_2$: C, 33.47; H, 2.81; N, 4.34; found: C, 33.45; H, 2.62; N, 4.53.

2,2-Dibromo-*N*-(2-methoxyphenyl)acetamide (3f)

Colorless solid; mp 119.4–120.6 °C; IR (KBr) ν_{max}: 3273, 1691, 1600, 1544, 1442, 1298, 1174, 968, 860, 810, 756 cm^{-1}; ^{1}H NMR ($CDCl_3$, 400 MHz) δ 8.85 (s, 1H), 8.26–8.24 (d, *J* = 8.0 Hz, 1H), 7.12–7.08 (t, *J* = 8.0 Hz, 1H), 7.96–7.00 (t, *J* = 8.0 Hz, 1H), 6.92–6.90 (d, *J* = 8.0 Hz, 1H), 5.92 (s, 1H), 3.92 (s, 3H); ^{13}C NMR ($CDCl_3$, 100 MHz) δ 161.6, 148.4, 126.4, 125.0, 121.1, 119.6, 110.2, 56.0, 36.9; EIMS *m*/*z* (%): 149.00 (100.00), 322.70 (9.22); Anal. calcd for $C_9H_9Br_2NO_2$: C, 33.47; H, 2.81; N, 4.34; found: C, 33.29; H, 2.99; N, 4.52.

2,2-Dibromo-*N*-(2,4-dichlorophenyl)acetamide (3g)

Colorless solid; mp 163.7–165 °C; IR (KBr) ν_{max}: 3273, 1691, 1600, 1544, 1442, 1298, 1174, 968, 860, 810, 756 cm^{-1}; ^{1}H NMR ($CDCl_3$, 400 MHz) δ 8.76 (s, 1H), 8.35 (s, 1H), 7.34–7.32 (d, *J* = 8.0 Hz, 1H), 7.10–7.08 (d, *J* = 8.0 Hz, 1H), 5.93 (s, 1H); ^{13}C NMR ($CDCl_3$, 100 MHz) δ 162.0, 134.2, 133.7, 129.8, 125.8, 121.8, 121.1, 36.1; EIMS *m*/*z* (%): 325.65 (100.00), 362.55 (17.56); Anal. calcd for $C_8H_5Br_2Cl_2NO$: C, 26.55; H, 1.39; N, 3.87; found: C, 26.61; H, 1.54; N, 3.91.

2,2-Dibromo-*N*-*p*-tolylacetamide (3h)

Pale yellow oil; IR (KBr) ν_{max}: 3273, 1691, 1600, 1544, 1442, 1298, 1174, 968, 860, 810, 756 cm^{-1}; ^{1}H NMR ($CDCl_3$, 400 MHz) δ 8.12 (s, 1H), 7.41–7.39 (d, *J* = 8.0 Hz, 2H), 7.16–7.14 (d, *J* = 8.0 Hz, 2H), 5.92 (s, 1H), 2.32 (s, 3H); ^{13}C NMR ($CDCl_3$,

100 MHz) δ 162.0, 135.8, 135.3, 129.6, 120.2, 36.8, 20.9; EIMS *m/z* (%): 134.00 (100.00), 306.70 (28.43); Anal. calcd for $C_9H_9Br_2NO$: C, 35.21; H, 2.96; N, 4.56; found: C, 35.08; H, 3.09; N, 4.81.

2,2-Dibromo-*N*-(4-ethoxyphenyl)acetamide (3i)

Colorless solid; mp 135.8–137.1 °C; IR (KBr) ν_{max}: 3273, 1691, 1600, 1544, 1442, 1298, 1174, 968, 860, 810, 756 cm^{-1}; ^1H NMR ($CDCl_3$, 400 MHz) δ 8.04 (s, 1H), 7.42–7.40 (d, *J* = 8.0 Hz, 2H), 6.88–6.86 (d, *J* = 8.0 Hz, 2H), 5.92 (s, 1H), 4.03–3.98 (d, *J* = 8.0 Hz, 2H), 1.41–1.37 (t, *J* = 8.0 Hz, 2H); ^{13}C NMR ($CDCl_3$, 100 MHz) δ 162.01, 156.7, 130.1, 128.4, 121.9, 114.9, 63.7, 14.7; EIMS *m/z* (%): 108.00 (100.00), 336.75 (72.27); Anal. calcd for $C_{10}H_{11}Br_2NO_2$: C, 35.64; H, 3.29; N, 4.16; found: C, 35.66; H, 3.11; N, 4.33.

2,2-Dibromo-*N*-(2,4-dimethoxyphenyl)acetamide (3j)

Colorless solid; mp 137.5–138.9 °C; IR (KBr) ν_{max}: 3273, 1691, 1600, 1544, 1442, 1298, 1174, 968, 860, 810, 756 cm^{-1}; ^1H NMR ($CDCl_3$, 400 MHz) δ 8.65 (s, 1H), 8.13–8.11 (d, *J* = 8.0 Hz, 2H), 6.48–6.46 (d, *J* = 8.0 Hz, 2H), 5.91 (s, 1H), 3.88 (s, 3H), 3.78 (s, 3H); ^{13}C NMR ($CDCl_3$, 100 MHz) δ 161.3, 157.4, 149.8, 120.4, 120.4, 103.9, 98.7, 56.0, 55.5, 37.0; EIMS *m/z* (%): 192.90 (100.00), 352.91 (89.82); Anal. calcd for $C_{10}H_{11}Br_2NO_3$: C, 34.02; H, 3.14; N, 3.97; found: C, 34.11; H, 3.07; N, 4.11.

2,2-Dibromo-*N*-(4-chloro-2,5-dimethoxyphenyl)acetamide (3k)

Colorless solid; mp 133.7–134.8 °C; IR (KBr) ν_{max}: 3273, 1691, 1600, 1544, 1442, 1298, 1174, 968, 860, 810, 756 cm^{-1}; ^1H NMR ($CDCl_3$, 400 MHz) δ 8.81 (s, 1H), 8.07 (s, 1H), 6.93 (s, 1H), 5.92 (s, 1H), 3.88 (s, 3H), 3.87 (s, 3H); ^{13}C NMR ($CDCl_3$, 100 MHz) δ 161.7, 149.2, 142.5, 125.6, 117.4, 112.6, 104.7, 56.8, 56.7, 36.6; EIMS *m/z* (%): 386.60 (100.00); Anal. calcd for $C_{10}H_{10}Br_2ClNO_3$: C, 31.00; H, 2.60; N, 3.62; found: C, 31.13; H, 2.55; N, 3.73.

2,2-Dibromo-*N*-methylacetamide (3l)

Orange oil; IR (KBr) ν_{max}: 3273, 1691, 1600, 1544, 1442, 1298, 1174, 968, 860, 810, 756 cm^{-1}; ^{1}H NMR ($CDCl_3$, 400 MHz) δ 6.50 (s, 1H), 5.81 (s, 1H), 2.92 (s, 3H); ^{13}C NMR ($CDCl_3$, 100 MHz) δ 164.2, 27.8, 23.9; EIMS *m*/*z* (%): 58.05 (100.00), 230.65 (3.49); Anal. calcd for $C_3H_5Br_2NO$: C, 15.61; H, 2.18; N, 6.07; found: C, 15.42; H, 2.21; N, 6.19.

References

1. Yang, Y. C.; Shang, P. H.; Cheng, C. M.; Wang, D. C.; Yang, P.; Zhang, F.; Li, T. W.; Lu, A. J.; Zhao, Y. F. *Eur. J. Med. Chem.* **2010**, *45*, 4300–4306.
